# Artificial intelligence and machine learning in pain research: a data scientometric analysis

**DOI:** 10.1097/PR9.0000000000001044

**Published:** 2022-11-03

**Authors:** Jörn Lötsch, Alfred Ultsch, Benjamin Mayer, Dario Kringel

**Affiliations:** a Goethe—University, Institute of Clinical Pharmacology, Frankfurt am Main, Germany; b Fraunhofer Institute for Translational Medicine and Pharmacology ITMP, Frankfurt am Main, Germany; c DataBionics Research Group, University of Marburg, Hans—Meerwein-Straße, Marburg, Germany

**Keywords:** Data science, Machine learning, Biometrics, Knowledge discovery, Pain, Precision medicine

## Abstract

The collection of increasing amounts of data in health care has become relevant for pain therapy and research. This poses problems for analyses with classical approaches, which is why artificial intelligence (AI) and machine learning (ML) methods are being included into pain research. The current literature on AI and ML in the context of pain research was automatically searched and manually curated. Common machine learning methods and pain settings covered were evaluated. Further focus was on the origin of the publication and technical details, such as the included sample sizes of the studies analyzed with ML. Machine learning was identified in 475 publications from 18 countries, with 79% of the studies published since 2019. Most addressed pain conditions included low back pain, musculoskeletal disorders, osteoarthritis, neuropathic pain, and inflammatory pain. Most used ML algorithms included random forests and support vector machines; however, deep learning was used when medical images were involved in the diagnosis of painful conditions. Cohort sizes ranged from 11 to 2,164,872, with a mode at n = 100; however, deep learning required larger data sets often only available from medical images. Artificial intelligence and ML, in particular, are increasingly being applied to pain-related data. This report presents application examples and highlights advantages and limitations, such as the ability to process complex data, sometimes, but not always, at the cost of big data requirements or black-box decisions.

## Abstract 2

Machine learning applications are rapidly increasing in pain research and are applied to patient data, while they seem to be little used in preclinical research.

## 1. Introduction

The collection of increasing amounts of data in health care and the trend towards digitalized medicine do not leave out the care for pain patients and the research for improved pain treatments. Examples of larger amounts of pain-related data include so-called "omics" information, medical images, or more nuanced information related to clinical phenotype, resulting from the recognition that pain is a complex characteristic that can only be partially captured by intensity.^26^ The expectation of this growing data set is better and more precise therapy for pain. However, large data sets with complex composition, occasionally combining multiple modalities from numerical data to images or patient-generated descriptions of symptoms, pose problems in their analysis, which ultimately aims to extract information from the data that can be transformed into knowledge about pain.

The developments in informatics and data analysis that have paralleled data collection in recent decades are available for analysis of pain-related data. Although artificial intelligence (AI) is widely discussed, the most relevant set of methods includes machine learning, which can be considered a subfield of AI and is its main methodological foundation to date. Definitions are subject to debate, but AI can be described as a branch of computer science that deals with the automation of human activities that are generally classified as intelligent behavior.^60^ These activities include understanding human language, representing and using knowledge, reasoning, planning, problem solving, risk assessment, and learning from experience or to extract information from the data that are useful for deriving new knowledge. Machine learning is currently the most popular method of AI and can be described as a set of methods that can automatically detect pattern, such as subgroups in the data and then use the detected patterns to assign future data to the correct subgroup.^9,15,68^

**Figure 1.**
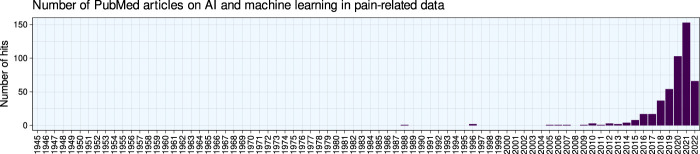
Bar chart of the number of publications per year. The figure has been created using the software package R (version 4.2.0 for Linux; https://CRAN.R-project.org/)^73^ and the library “ggplot2” (https://cran.r-project.org/package=ggplot2).^93^

Machine learning is increasingly being used in pain research, according to a query of the PubMed database (Fig. 1). In this scientometric analysis, after a brief recapitulation of the principles and main workflow of machine learning, the publications on the application of machine learning methods and AI will be summarized in the current context of pain research. Commonly used machine learning and artificial intelligence methods and the pain settings most frequently addressed with these methods to date will be highlighted, followed by a brief discussion of implications of their use in research and care for patients with pain.

### 1.1. Workflow and main methods of machine learning–based data analysis

**Figure 2.**
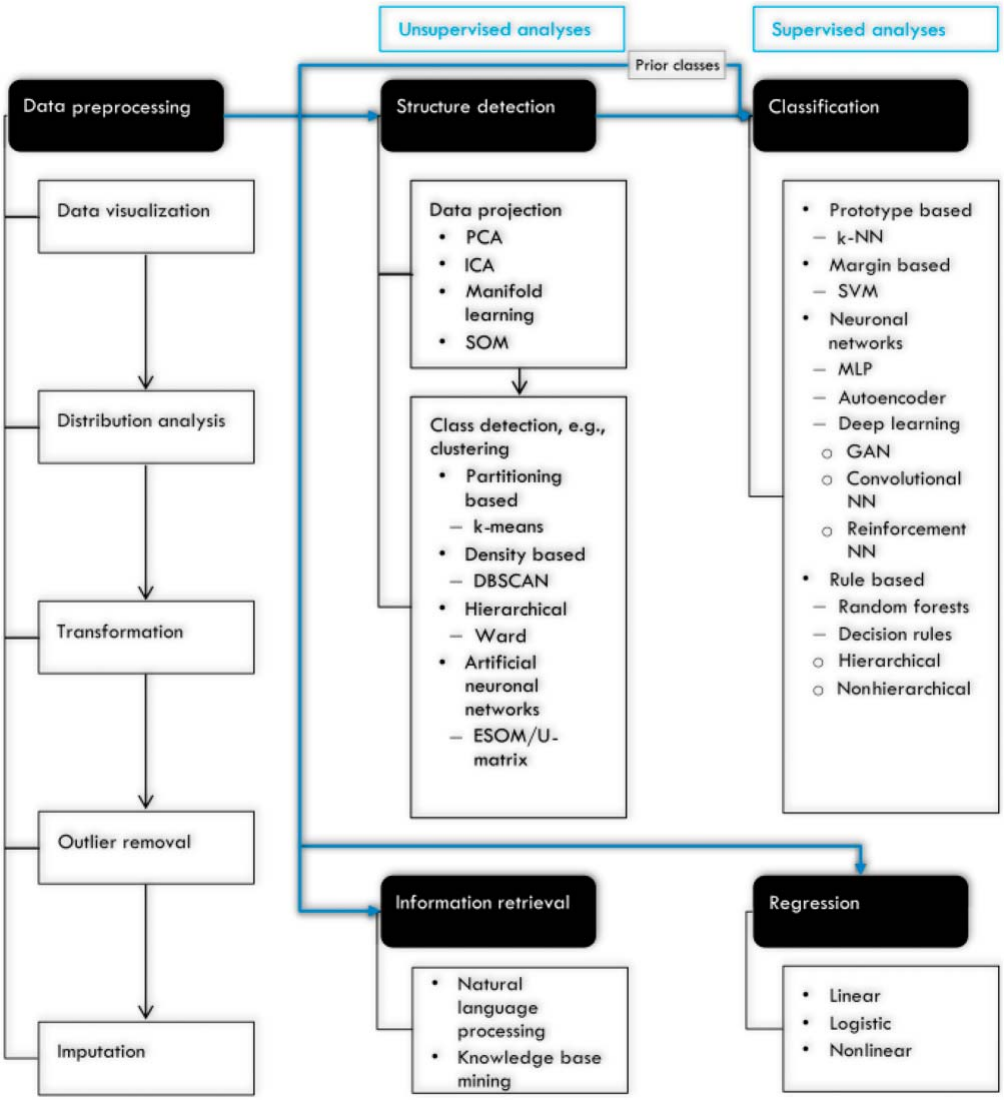
Frequent workflow of analyzing a data set with machine learning. Preprocessed data can be subjected to structure detection including subgroup or class detection, or directly when previous classes are known or classes are not addressed, to classification, regression, or information retrieval, depending on the characteristics of the data and the actual analytical goals. All steps do not always have to be present. The selection of the example methods has been adapted to the mentions in this report. Classification methods can be applied to predetermined classes (previous classes) of interest, eg, patients vs controls, or a class structure can be detected using machine learning, eg, clusters or subgroups of patients that are not known at the outset. Types and subtypes of methods are connected by lines. If a standard sequence of steps is relevant, arrows are drawn. Convolutional NN, convolutional neural network; DBSCAN, density-based spatial clustering of applications with noise; ESOM, emergent self-organizing map; GAN, generative adversarial network; ICA, independent component analysis; k-NN, k-nearest neighbors; MLP, multilayer perceptron; PCA, principal component analysis; SOM, self-organizing map; SVM, support vector machine; U-matrix, unified distance matrix. The figure was created using Microsoft PowerPoint (Redmond, WA) on Microsoft Windows 11 running in a virtual machine powered by VirtualBox 6.1.34 (Oracle Corporation, Austin, TX) as a guest on Linux and then further modified with the free vector graphics editor “Inkscape” (version 1.2 for Linux, https://inkscape.org/).

Analyzing a data set with machine learning follows a workflow (Fig. 2) that begins with preprocessing the data. Main tasks of machine learning include the discovery of structures in the data, which is performed using so-called unsupervised methods (see below), and the acquisition of the ability to assign the correct label to a case in the data set based on numerical information about certain properties of the data instance, eg, a clinical diagnosis, which is performed using so-called supervised methods. Other applications include exploring dependencies between variables and outcomes through regression analysis. Further tasks include describing these structures in an understandable way based on the given features, ie, the variables in the data set.

Extracting knowledge from data can also be performed through natural language processing, such as in the systematic analysis of patient satisfaction after total joint arthroplasty using patient comments^67^ or by mining large databases and combining the knowledge they contain from different sources. For example, functional genomics-based drug discovery^51^ using the Gene Ontology knowledge base^2^ at https://www.geneontology.org/combined with the DrugBank database^94^ at https://go.drugbank.com. The following sections briefly describe some of the major approaches; additional methods are addressed with summaries of the application of machine learning to pain-related data, based on the frequency of their use in this context.

### 1.2. Data preprocessing

Data preprocessing is a crucial step in machine learning and should start with visualization of the data because visual inspection by an expert still seems to be the most direct way to detect anomalies such as implausible values in biomedical data and to improve the discussion of the data between the data scientist and the medical expert. Data preprocessing includes, among others, the analysis of the distribution of the variables, appropriate transformations, followed by the removal of outliers and the imputation of missing values. For the latter, in addition to classical univariate methods such as substitution by specific scalars (eg, mean or median), there are machine learning–based methods that replace missing values with values learned from the available data in a multivariate fashion, including distance-based models, such as k-nearest neighbors^11^ and classification and regression trees (CART),^6^ or ensemble models, such as random forests,^5,31^ which we have recently summarized elsewhere.^62^ In addition, data processing for machine learning may involve specific transformation methods that optimize the data structure for distance-based subgroup or cluster detection, such as pooled variable scaling for cluster analysis^74^ or data transformation optimized for Euclidean distance.^89^

### 1.3. Structure discovery

**Figure 3.**
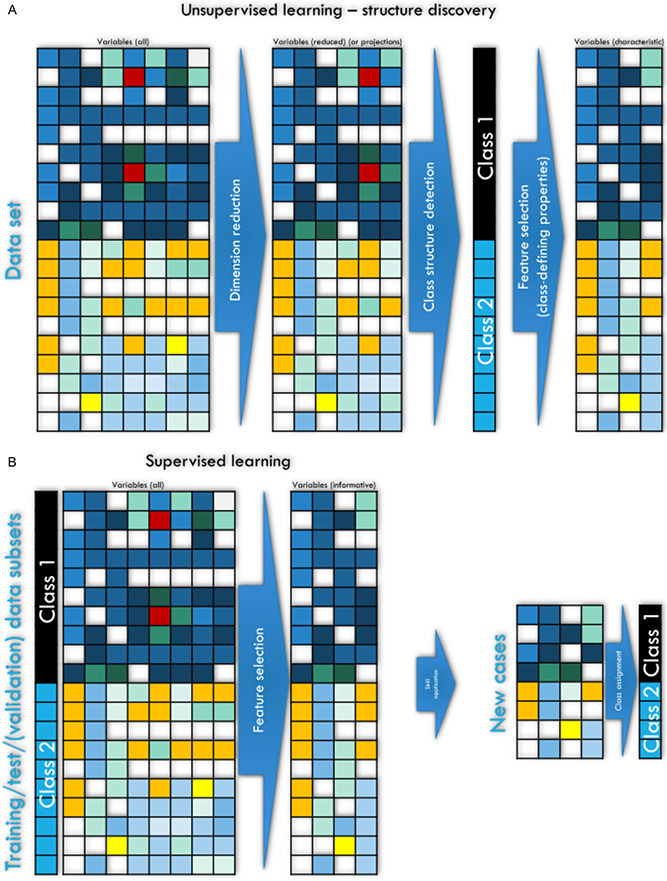
Schematic overview of the main types and implementations of machine learning (ML). (A and B) Workflow of ML approaches. ML approaches to (A) unsupervised structure discovery, including (left part) the unsupervised detection of relevant structures in the data and the identification of key variables that characterize the detected subgroup structure (feature selection), and (B) machine learning approaches aimed at developing automated diagnostic tools or biomarkers through supervised training of algorithms to assign a case to the correct subgroup and to identify the relevant variables informative to this task (feature selection) to be used with new data that the algorithm has not seen during training, with the task of making a class assignment such as a diagnosis. The matrix heat plots have been intentionally created to symbolize different expressions of features (columns) among 2 classes of individuals (rows), with darker colors symbolizing higher values in the variables. The figure was created using Microsoft PowerPoint (Redmond, WA) on Microsoft Windows 11 running in a virtual machine powered by VirtualBox 6.1.34 (Oracle Corporation, Austin, TX) as a guest on Linux and then further modified with the free vector graphics editor “Inkscape” (version 1.2 for Linux, https://inkscape.org/).

**Figure 4.**
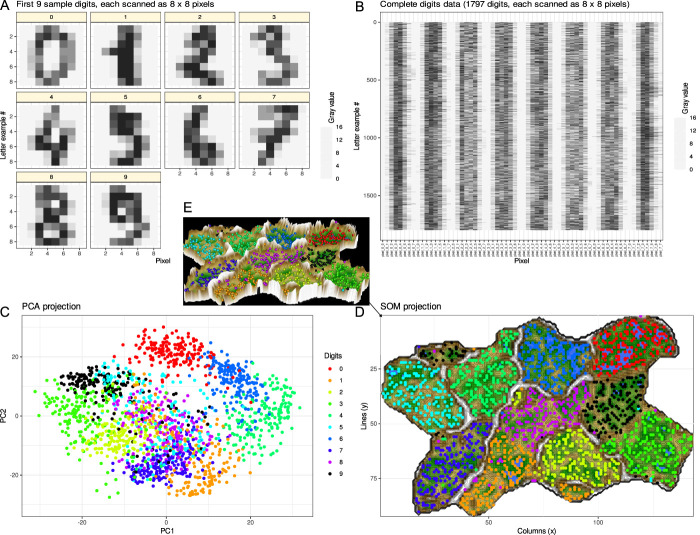
Example of data structure detection after a data projection as a frequent first step. A generic data set was used containing 8 × 8 pixel scans of handwritten digits 0, …, 9 from the data set collection of the Python package “scikit-learn” (https://scikit-learn.org/stable/).^71^ The pixels were numerically converted to gray values. (A) First 10 digits in the data set given as an example. (B) Matrix plot of the complete data set of scanned handwritten digits from 1,797 individuals. The 64 pixels of which each digit is comprised are arranged in rows of 64 numerical gray-values. The complete data set has little meaningful structure among the gray values, analogous to many biomedical data sets, including pain-related data, where measurements from patients are included instead of gray values but an immediate subgroup structure cannot be seen. The task in detecting a data structure is to identify classes/subgroups/clusters in the data set, such as identifying in this example that the data set in panel B contains gray values of 10 classes of handwritten digits. (C) Principal component analysis (PCA)-based projection of the data set onto a 2-dimensional plane using principal component (PC) 2 vs PC1. The dot plot is color coded for the classes, ie, the different digits. On the PCA projection, the 9 different digits are partially separable, such as “0” (red) forming a separate cluster at the top, suggesting that the projection enhances the detection of a class structure in the data set that was obscured in panel B. However, without knowing the ground truth as in the present example, it would be difficult to see that the data set contains 9 different classes. (D) Alternative data projection using machine learning in the form of a self-organizing map of artificial neurons. The panel shows results of projection of data onto an emergent self-organizing map (ESOM^85,90^) neurons, providing a 3-dimensional U-matrix visualization of distance-based structures of the gray values after projection of the data points onto a toroid grid of 9,000 neurons where opposite edges are connected using a Gauss-formed neighborhood function and 25 training epochs for the SOM. The dots represent the so-called “best-matching units” (BMUs), ie, neurons on the grid that after ESOM learning carried a data vector that was most similar to a data vector of a sample in the data set. The U-matrix visualization was colored as a top view of a topographic map with brown (up to snow-covered) heights and green valleys with blue lakes. Watersheds indicate borderlines between 2 different clusters separated by the white “mountain ridge” at the left of the U-matrix. (E) Separation between the classes is better visible in a 3D presentation of the U-matrix in panel D. The ESOM projection detected most of the classes separately; however, the separation was also not perfect and with “9” seems to be split into 2 classes, which might hint at indeed to main versions of writing “9”. In this example, this was not further analyzed, but in biomedical data, this might lead to the discovery of a so far unknown subgroup. The figure has been created using the software package R (version 4.2.1 for Linux; https://CRAN.R-project.org/)^73^ and the library “ggplot2” (https://cran.r-project.org/package=ggplot2)^93^ and our library “Umatrix” (https://cran.r-project.org/package=Umatrix).^53^

One of the most important forms of machine learning aims at extracting information and ultimately knowledge from data. An important task in this context is the discovery of structures in the data, such as subgroups or other useful insights that were not directly obvious. The computer-aided detection of structures in data, eg, classes or subgroups such as subtypes of diseases, is often referred to as “unsupervised machine learning” (Fig. 3). This means that one cannot evaluate the learning success based on an a priori known result, eg, a predefined subgroup structure, because finding such a structure defined by the (dis-) similarities within the data is the actual task of the analysis. Thus, unsupervised machine learning strategies involve techniques that allow the user to evaluate the data after sufficient preparation without any previous knowledge. The quality of the achieved subgroup separation/cluster solution is therefore mainly quantified by formal criteria or its usefulness. Examples of algorithms used for clustering include k-means centroid localization based on Euclidian distance,^61^ Ward hierarchical^92^ clustering, density-based spatial clustering of applications with noise (DBSCAN),^66^ or self-organizing maps (SOMs).^38^ Of note, as mentioned previously,^58^ there are 2 types of SOMs, with one using a small number of neurons that are identified with clusters and a second type of which one feature is the usage of a large number of neurons up to thousands (for details about the number of neurons, refer to 53), termed emergent SOM (ESOM), where emergence, ie, the appearance of higher-level structures due to microscale interactions, can be observed by looking at structures like ridges or valleys consisting of groups of neurons.^87^ An example is provided in Figure 4.

### 1.4. Dimensionality reduction and feature selection

Reducing data dimensionality before training machine learning–based classifiers by filtering out uninformative or redundant variables is a standard procedure to limit computational load, simplify models for easier interpretation by field researchers, improve data compatibility with classifier models, or avoid other dimensionality drawbacks. This process is referred to as feature selection^27^ and is a preliminary step of the training of supervised algorithms. The reason for mentioning feature selection with unsupervised algorithms is its possible use for structure discovery. The approach assumes that if a classifier can be trained to assign a patient to the correct class better than by guessing, then the variables needed by the classifier to accomplish this task contain relevant information about the addressed class structure. In this way, the most informative variables can be identified. In this use of feature selection, creating a powerful classifier is not the final goal, but feature selection takes precedence over classifier performance. It is worth noting that in multivariate applications, the best predictors may not be variables that differ significantly between classes, or may even be variables that lack statistical significance.^49^

Approaches to feature selection^80^ include both univariate and multivariate methods, in which informative features can be obtained by, for example, recursive feature elimination or sequential feature selection. Particular implementations include the regression-based least absolute shrinkage and selection operators^81^ or make use of usually well-performing machine learning methods such as random forests^5,31^ and combine them with statistical tests as in the “Boruta” method^41^ or computed ABC analysis to obtain the informative features with item categorization.^59^

### 1.5. Regression and classification

The main tasks of the so-called “supervised” methods include classification and regression. In regression, the target variable is present in a numerical scale and various regression models are fitted to the data to obtain the best hyperplane passing through the data points. In this way, the numerical value of the target variable can be predicted for future cases unseen during fitting of the regression model. In classification, the target variable is present as a class label, eg, men or women. The task is to assign a set of features (variables and properties) to a particular class label of the instances of the data set (eg, patients or medical images). The class may be present as a previous classification, eg, patients vs controls, or may be the result of the unsupervised analysis mentioned above, eg, cluster membership. ML classification methods use examples of data to tune algorithms to optimize the prediction or classification of new, unseen cases (Fig. 3). The machine acquires the ability to correctly label a case in the data set based on information about certain properties of the data instance, eg, pain intensity in a patient, concentration of a molecule in the blood, etc. Learning is performed by repeating the task with increasing success. This type of machine learning is referred to as supervised because the learning success is evaluated based on an a priori known outcome, such as diagnostic classes. Important examples of such algorithms are support vector machines,^10^ random forests,^5,31^ k-nearest neighbors,^11,19^ or convolutional neural networks.^46^

Supervised methods are powerful in assigning cases to the correct group based on given variables. They mimic the clinical diagnosis made based on various medical information from the patient, such as interviews, questionnaires, laboratory tests, medical images, etc. However, the training of supervised algorithms requires previous knowledge of the (diagnostic) class structures in the data set. Supervised algorithms fall therefore short if subgroups of patients (stratification) are not known. This knowledge can be obtained using unsupervised algorithms described in the following section.

### 1.6. Scientometric analyses of publication activities on machine learning in pain research

#### 1.6.1. Query of machine learning approaches used in pain research

Programming was performed in the R language^34^ using the R software package,^73^ version 4.2.0 for Linux, available free of charge from the Comprehensive R Archive Network (CRAN) at https://CRAN.R-project.org/. The R code with which this scientometric analysis on machine learning has been created is available at https://github.com/JornLotsch/AI4pain.

The use of AI and machine learning in pain research was queried from the PubMed database at https://pubmed.ncbi.nlm.nih.gov/on May 6, 2022. The search was performed in the classical way directly on the PubMed website and again using the R library “RISmed” (https://cran.r-project.org/package=RISmed),^39^ using the search string given in Box 1. Subsequently, the abstracts of the retrieved articles were filtered by automated search to see whether they contained the queried terms in the intended way, eg, “artificial intelligence” instead of “intelligence” and “artificial” separated from each other in the text. Only reports that passed this filtering were retained.

Text box 1. **Search string used in an automated query of the PubMed database**

(https://pubmed.ncbi.nlm.nih.gov) using the R library “RISmed” (https://cran.r-project.org/package=RISmed).^39^ Of note, adding “ai” to the search string was not useful because >8,000 hits were then found, most of which simply contained the 2 letters in that order, as in “pain”. Because scientific journals require that all abbreviations be spelled out the first time they are used, including in the abstract, the absence of “ai” in the search string containing “artificial intelligence” is not a limitation.

((machine-learning) OR (machine learning) OR (machine-learned) OR (machine learned) OR (artificial intelligence) OR (explainable AI) OR (explainable artificial intelligence) OR (XAI) OR (knowledge discovery) OR (deep learning)) AND ((chronic) OR (persisting) OR (persistent) OR (lasting) OR (neuropathic) OR (nociceptive) OR (nociplastic) OR (mixed) OR (neurogenic) OR (back) OR (neck) OR (migraine) OR (arthritis) OR (osteoart*) OR (joint) OR (rheumatic) OR (orchialgia) OR (inflammatory) OR (musculoskeletal) OR (muscle) OR (visceral) OR (widespread) OR (somatoform) OR (fibromyalgia) OR (cancer) OR (postoperative) OR (perioperative)) AND ((pain) OR (painful) OR (analgesi*)) NOT (review[Publication Type])

The above search resulted in 475 hits. The earliest articles were published in 1988. Their research topics included computer-aided diagnosis of low back pain compared with physician diagnosis,^64^ an expert system for medical education of patients with low back pain,^69^ and a proposal to use a robotic system for nonpainful dermatologic treatment of port wine stain birthmarks exposure.^78^ However, 79% of the retrieved studies were published since 2019 and 68% since 2020. Thus, the use of ML for pain has increased rapidly over the last decade (Fig. 1).

### 1.7. Frequent machine learning methods used with pain-related data

According to the mention in the abstracts of the retrieved articles found, regression, deep learning, random forests, support vector machines, and hierarchical clustering were most frequently applied to pain-related data (Table 1). Regression models are commonly used for statistical analyses of biomedical data. They are implemented in virtually all software packages used in the present research field and should be sufficiently well known. It should be noted, however, that in the abstracts the methods used are often not fully listed. The search results may therefore be not complete, unlike the examples in the paragraphs above which are based on the main body of the texts including the figures. Nevertheless, the most common methods are consistent with the general observation of common ML approaches for biomedical data.

**Table 1:** Types of machine learning methods named in abstracts of the 475 reports on artificial intelligence (AI) and machine learning in pain research queried in PubMed.

Method	Hits
Regression	94
Deep learning	68
Random forests	67
Support vector machines	55
Hierarchical clustering	47
Convolutional neural networks	25
k-nearest neighbors	18
Natural language processing	12
Principal component analysis (PCA)	9
k-means clustering	8
Self-organizing maps (SOMs)	7
(Multilayer) perceptron	6
Independent component analysis	4
Autoencoder	3
Reinforcement learning	2
Generative adversarial networks (GANs)	1
Density-based spatial clustering of applications with noise (DBSCAN)	1

The numbers are the result of a full-text search of the abstracts for the keywords listed in the table.

### 1.8. Unsupervised methods

Unsupervised machine learning strategies include techniques that allow users to evaluate data without previous knowledge after sufficient preparation. Unsupervised techniques help decompose complex data structures for further learning or provide insights that were not obvious at first glance. Based on mentions in the abstracts of the articles found, clustering seems to be the most commonly used unsupervised analysis of pain-related data (Table 1). Hierarchical clustering, often implemented as the Ward method,^92^ and partition-based clustering implemented as k-means clustering^61,84^ were among most commonly used methods. Although both methods are implemented in most statistical software packages, including user-friendly point-and-click solutions, using them in a formal way without realizing that clustering algorithms typically specify a shape model for the structure of a cluster can result in spurious clusters. In k-means clustering, for example, an iterative procedure partitions the cases into k clusters such that each observation belongs to the cluster with the closest center. This implicitly assumes a hyperspheric cluster form, which can lead to incorrect cluster associations of samples or imposition of cluster structures that are not present in the data.^88^ This can be avoided if other methods such as emergent self-organizing feature maps (ESOMs)^38^ are used in combination with the so-called U-matrix^86^ (Fig. 4). Although k-means and classical hierarchical clustering methods search for clusters over distances between data points and are sensitive to the scaling of the data, as for example in the case of Euclidean distance, which induces a breakpoint for distances within (inner) clusters vs distances between (inter) clusters at a value of 1 due to the squaring function in its definition, ESOMs can additionally obtain clusters over the density of data points in high-dimensional space.^85^ Distances between data points are also considered with density-based spatial clustering of applications with noise (DBSCAN) as another recent clustering method.^18^

One of the most commonly used classical data projection methods for dimensionality reduction and improving structure recognition is principal component analysis (PCA),^70^ which was among the most frequently mentioned unsupervised methods in the abstracts of retrieved articles (Table 1). It uses a rotation of the data to project the data onto a subspace of so-called principal components. The first principal component has the largest possible variance in the data. Each subsequent orthogonal component is selected according to the largest possible remaining variance. Although PCA is part of the routine data analysis workflow and was probably even used more frequently in the articles found, without mention in the abstract, alternative projection methods should usually be considered, as shown in a generic example data set comparing PCA projection with ESOM (Fig. 4). Moreover, the abbreviation “PCA” is used ambiguously in scientific publications on pain, eg, for “principal component analysis”^32,70^ as a data analysis method and also for “patient-controlled analgesia” as a drug delivery method. In 3 reports of machine learning results in pain research, PCA was used only in the latter meaning, and Table 1 was corrected accordingly after a negative search for “principal component analysis” in the full text.

### 1.9. Supervised methods

Apart from regression analysis, different types of classifiers were most commonly used in supervised analysis of pain-related data, with “deep learning” (for an overview, refer to 14) being the most frequently mentioned. This refers to a set of algorithms with different architectures that have in common that they use artificial neural networks to uncover the internal structures of the data, following the idea of constructing a network of firing computational units resembling neuron branches that sequentially perform mathematical operations and pass the data from layer to layer.^29,65^ Technically, artificial neural networks are based on linear combinations, using a scalar input that, with the help of a compiled model consisting of activation function steps and optimization procedures, undergoes various transformations as it passes through, for example, a multilayer perceptron, and, at the end, provides an output, such as a class label.^82^ The use of different activation functions (eg, a sigmoidal or a rectifying linear unit function) allows computations to be performed through the network.^13,29,30^ Typical architectures include convolutional neural networks (CNNs). Convolutional neural networks are typically used for image segmentation and classification problems based on transformation of imaging data like U-net for example.^76^ Some semisupervised methods rely on autoencoders^79^ and are used to train classifiers based on hidden feature exploration.^96^ Other self-supervised learning architectures can be connected to reinforcement learning (RL) (for a review, refer to 35). Compared with regular supervised machine learning, RL does not rely on showing input and output but on the experience an agent is facing towards its environment. Reinforcement learning is based on a “trial-and-error” reward learning performed through an agent subjected to a set of discrete states and actions.

Some of the deep learning architectures belong to the so-called generative ML models, which aim to learn the true data distribution of a training set to generate new data points that validly extend the existing data set. For example, generative adversarial networks (GANs^12^) have been shown to be extremely powerful for certain tasks such as the GAN in image processing.^12^ Generative adversarial networks consist of 2 main parts, the generative network and the discriminative network. Although the generative network learns from the data distribution and produces new valid data, the discriminative network has the task of distinguishing the generated data from the real data. Through mutual interaction, both parts successively improve their performance. In a pain context, GANs have been used for automated diagnosis of metacarpophalangeal synovitis from musculoskeletal ultrasound images taken from patients with rheumatoid arthritis.^8^ The data included physician-made synovitis grading and 446 ultrasound images from 446 patients. A high-resolution GAN was developed and trained to assign patients to the correct synovitis grading, ie, the problem was transformed into a classification task. The images were decomposed into 7,152 data set instances in the training set and 1,791 data set instances in the validation subset drawn from the whole data set. Within the GAN, the generator analyzed the features of the images, downsampled them and generated new images that were passed to the discriminator part of the GAN. The GAN achieved 83% accuracy on the task to grade the synovitis from the ultrasound images and outperformed 2 other types of deep learning neural networks that achieved 78% to 79% accuracy.

Further often used supervised algorithms that have gained importance in particular in non–image-based analyses and in smaller data set were random forests and support vector machines, which are generally known as well-performing algorithms applicable to a wide range of data. In random forest analysis,^5,31^ sets of different, uncorrelated, and often very simple decision trees are created with conditions on variables (features) as vertices and classes as leaves. Each tree in the random forest votes for a class, and the final classification assigned to a data point is obtained as the majority of these class votes. The number of trees ranges from about 100 to more than 2,000. Their complexity can also be controlled, often using the square root of the number of variables included as the default value. Support vector machines are ML classifiers which use kernel functions to assign data to given classes.^10^ Kernel functions calculate distances in a hyperspace where the original data are mapped into. This hyperspace is typically much higher dimensional (up to infinity) than the feature space of the data. In some cases, the classification is easier in the hyperspace than in the data's space.^28^

### 1.10. Pain settings addressed with machine learning methods

Machine learning algorithms have been trained to assign complex features to a known class of patients with pain or to identify risk factors and predict the severity and clinical course of pain. Representative examples where the ML approach has led to particularly relevant clinical advances are presented below, without describing all 475 references found. The most common types or causes of pain addressed with ML methods included (low back) pain, musculoskeletal pain, and osteoarthritis pain (Table 2). Less frequently cited, but with more than 10 reports each, are neuropathic and inflammatory pain, followed by widespread pain and fibromyalgia.

**Table 2:** Types and etiologies of pain cited in abstracts of the 475 reports on artificial intelligence (AI) and machine learning in pain research queried in PubMed.

Pain types	Hits	Pain duration	Hits	Clinical settings	Hits
Neuropathic	27	Chronic	195	Back pain	94
Nociceptive	20	Acute	46	Musculoskeletal	64
Nociplastic	0			Osteoarthritis	57
Mixed	0			Neuropathic	24
				Inflammatory	23
				Widespread	10
				Fibromyalgia	10
				Visceral	3
				Idiopathic	1

The numbers are the result of a full-text search of the abstracts for the keywords listed in the table.

### 1.11. Machine learning targeting medical images in the context painful diseases

High-performance machine learning algorithms were often trained with medical images acquired in the context of a painful condition, ie, pain was not the direct target when learning such approaches for low back pain or arthritis pain. The pain context was obtained through additional analyses. Therefore, these reports are included, however, in a separate subchapter. However, there are more complex forms of pain, and the International Association for the Study of Pain definition does not speak of “images” but of subjective perception and even of just possible tissue damage.

### 1.12. Machine learning approaches to imaging in the context of low back pain

In the context of low back pain, nerve ingrowth may occur along granulation tissue in disk fissures. This has been used to classify those fissures and the pain caused by a discography. The data set included magnetic resonance and computed tomography images of 86 pain-positive discograms and a similar number of intraindividual control disks, acquired from 30 patients.^43^ A random forest algorithm was trained in a Python^91^ programming environment to differentiate between individual disks with fissures that did or did not extend into the outer layers of the annulus fibrosus and between disks with positive and negative pain provocation on discography. The image diagnostic task was successfully completed with a very high hit precision of 99% on average in repeated tasks and 97% accuracy. However, pain was only captured at 71% precision and 69% accuracy.

Although the amount of data was limited and only a single algorithm was used, the results show that machine learning performs better in discriminating medical images based on data such as contrast levels, edges, gray tones, etc., than in trying to discriminate clinical function parameters such as pain, which are rarely expressed in such images. It is therefore not surprising that medical imaging also features prominently in many reports on machine learning and AI-based approaches to pain management. However, it should be remembered that tissue damage and pain are not identical.^83^ Therefore, it cannot be assumed that an accurate AI-based diagnosis of the degree of tissue damage from medial images can be translated into a similarly accurate diagnosis of pain, but this requires training with more specific information about the pain itself.

### 1.13. Machine learning approaches to imaging in the context of osteoarthritis pain

A deep learning approach is used to quantify the severity of osteoarthritis using radiographs of the knee to predict patients' perception of pain.^72^ Clinical and radiographic data were analyzed from the Osteoarthritis Initiative (OAI; https://nda.nih.gov/oai/). This is a multicenter longitudinal study of participants aged 45 to 79 years who had knee osteoarthritis or were at high risk for developing knee osteoarthritis. The data set was divided into a training group of 2,877 subjects and a validation group of 1,295 subjects with 25,049 and 11,320 individual observations, respectively. Previous classifications were available based on radiologists' assessment of images for radiological features of osteoarthritis, including summary measures of severity.

A convolutional neural network was trained to predict the pain-related osteoarthritis-specific Knee Injury and Osteoarthritis Outcome Score^77^ for each knee based on each radiograph. During training, the network was also tuned to predict 19 radiological features. The network was implemented in the Python programming language^91^ and run on an Nvidia graphics processing unit (Nvidia Corporation, Santa Clara, CA). The authors examined pain disparity, defined as a coefficient for the binary racial or socioeconomic group. That is, the difference in average pain between racial and socioeconomic groups when controlling for severity. The primary outcome was racial differences in pain between Black (16% of patients in the validation group) and non-Black patients (84%, of whom 97% were White). The previously unexplained proportion of racial differences in pain could be dramatically reduced. Compared with radiologist-measured severity scores, which accounted for only 9% (95% confidence interval, CI: 3%–16%) of racial differences in pain, algorithmic predictions accounted for 43% of differences, or 4.7-fold (95% CI: 3.2–11.8-fold), with similar results for lower-income and lower-educated patients. This suggests that much of the pain experienced by underserved patients is due to factors within the knee that are not reflected in standard radiographic measures of severity that a radiologist observes. The ability of the algorithm to reduce unexplained disparities was attributed to the racial and socioeconomic diversity of the training group. The authors concluded that because algorithmic severity measures better capture the pain of underserved patients and severity measures influence treatment decisions, algorithmic predictions could potentially address disparities in access to treatments such as arthroplasty.

### 1.14. Machine learning targeting pain and its relief

#### 1.14.1. Machine learning approaches to musculoskeletal and back pain

The use of intelligent algorithms has been proposed as a novel way to increase adherence to exercise therapy for chronic musculoskeletal disorders, which could improve clinical outcomes. For example, in n = 161 patients who were asked about their self-perceived benefits of using an artificial intelligence–equipped mobile app for self-treatment of chronic neck and back pain,^50^ an increase in time spent daily on therapeutic exercise was achieved. A decrease in the use of other interventions was found when using the artificial intelligence–equipped mobile app. The median Numerical Rating Scale score was 6 (interquartile range, IQR: 5–8) before and 4 (IQR: 3–6) after using the AI-embedded mobile app (95% CI: 1.18–1.81). Participants who used the AI-embedded mobile app for more than 6 months reported a reduction of 3 points. Low back pain as the target of a ML analysis was reported from a study that aimed at a biomarker based on MRI data on cerebral cortical thickness and fMRI data on cortical resting state compared with activation, acquired in n = 27 healthy volunteers and in patients with low back pain (n = 24).^44^ A support vector machine classifier achieved an accuracy of the low back pain diagnosis of 74.51% and an ROC-AUC = 0.787 (95% CI: 0.66—0.91).

#### 1.14.2. Machine learning approaches to postoperative pain

Prevention of persistent pain after surgery through early identification of high-risk patients is a clinical need. Using supervised ML, parameters predicting persistence of significant pain were identified in a cohort of 1,000 women followed up for 3 years after breast cancer surgery.^56^ Key subgroups of subjects with “persistent pain” and “nonpersistent pain” phenotypes were identified by a diagnostic tool consisting of 21 nonhierarchical rules that included psychological, demographic, and clinical factors. If at least 10 of the 21 rules were true, persistent pain was predicted with a cross-validated accuracy of 86% and a negative predictive value of approximately 95%. This can be used to identify patients for whom complex and time-consuming preventive therapies are unnecessary, allowing them to return to normal life more quickly after breast cancer surgery. Another analysis found that a short questionnaire with fewer than 10 psychological items provided nearly similar diagnostic accuracy in identifying patients with or at risk for persistent pain.^55^ This short questionnaire was developed by applying supervised machine learning feature selection, implemented as random forest analysis, followed by computed ABC analysis,^59^ to data obtained from standard questionnaires with more than 50 items.

### 1.15. Other pain-related research topics addressed by means of machine learning

Another application of ML is the recognition of pain-related facial expressions.^20^ Pain intensity was assessed in 1,189 adult patients undergoing surgery, and 2,971 photographs of facial expressions were included, most of which (44%) were taken when patients were not in pain, while only 13.5% of photographs were taken when patients were in severe pain of ≥7 on an 11-point Numerical Rating Scale. After splitting the data set into training/testing/validation subsets, a convolutional neural network was trained to predict pain intensity based on facial expression. This succeeded with only a modest 45% to 53% accuracy. However, this outperformed human experts who achieved an above average accuracy in predicting pain intensity of only 14.9%. The algorithm predicted severe pain based on facial expression with a sensitivity of 17.0% and a specificity of 41.1%.

Data mining in the Gene Ontology knowledge base using computational functional genomics methods enabled the reduction of a set of n = 540 genes, whose importance in pain has been demonstrated mainly by studies in transgenic mice, which queried in the Pain Genes database (http://www.jbldesign.com/jmogil/enter.html)^42^ to a subset of only 29 top-scoring genes that can be considered key genes in the functional biology of pain.^48^ This subset described the function of the entire set of pain-related genes, expressed as a polyhierarchy of Gene Ontology terms describing pain-related biological processes, with a recall and precision of 70% using only 5% of the original genes. Furthermore, knowledge discovery in the Gene Ontology database combined with recent findings on the genetic background of persistent pain in humans pointed to inflammatory and immune processes as a key mechanism in persistent pain.^40^ Specifically, 110 genes reportedly associated with the modulation of persistent pain in different clinical settings were clustered using so-called emergent self-organizing artificial neuron (ESOM) maps,^85^ ie, an extension of self-organizing maps^38^ by visualizing distances between data points to improve cluster detection.^88^ Subsequent computational enrichment analysis revealed 2 groups of biological processes, the immune system and nitric oxide signaling, emerged as key players in sensitization to persistent pain. This is highly biologically plausible and consistent with other lines of pain research. What is remarkable about this finding is that it was obtained purely using computational methods of knowledge discovery that have yielded results similar to elaborate clinical or preclinical studies.

### 1.16. Sample sizes in artificial intelligence and machine learning analyses of pain-related data

ML is often intuitively associated with large sample sizes, which leads to disregarding these methods for analyzing smaller data sets on the grounds. To some extent, this is not true. ML requires examples of the relevant structures in an empirical data set. This can be a very small set (“needles”). Adding more data (the “haystack”) can degrade the accuracy of a diagnostic tool. With careful selection of the data used for learning, even small samples can yield powerful diagnostic tools. In addition, data needs may be low for knowledge discovery in the sense described above, which focuses not on a biomarker or diagnostic tool but on identifying informative features for a pain-related phenotype.

**Figure 5.**
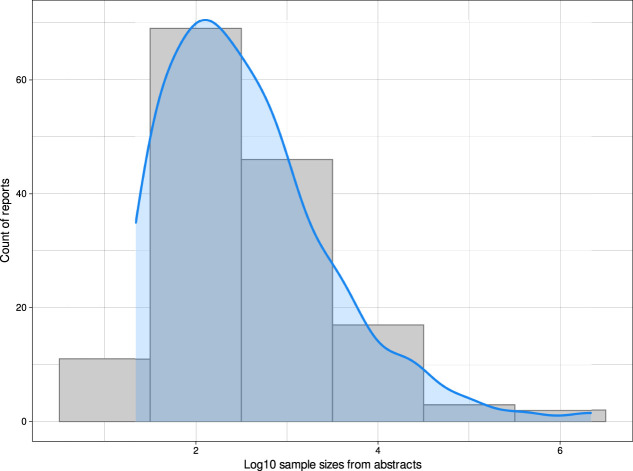
Sample sizes refer to reports on AI and machine learning in pain research. Sample sizes were manually extracted from the summaries of the 475 queried reports. The figure shows the distribution of sample sizes as a count density plot, drawn with the default settings of the built-in kernel density function of the R library “ggplot2”, overlaid with a histogram with binning for each power of 10. The figure has been created using the software package R (version 4.2.0 for Linux; https://CRAN.R-project.org/)^73^ and the library “ggplot2” (https://cran.r-project.org/package=ggplot2).^93^

Sample sizes were extracted manually from abstracts of articles found in PubMed by the above search. The work was performed independently by 2 authors and then cross-checked, with discrepancies discussed and corrected, followed by a final review by a third author. The reported case numbers ranged from 11 to 2,164,872, with a peak distribution at sample sizes of approximately n = 100 (Fig. 5). The smallest sample analyzed with ML was a data set of ambient sensor data in which pain-related behaviors of persons with chronic pain were sought.^23^ However, the analyzed data consisted of much more smart-home sensor data, so ML was not performed on only 11 data set instances. Other studies with small sample sizes were also imaging-based and the number of data instances analyzed was often much larger than the sample sizes of subjects included. The smallest ML approach where the analyzed data instances corresponded to the reported small sample size was predicting the length of hospital stays from nursing reports of n = 33 patients using a recurrent neural network long short-term memory with 2 hidden layers, which provided about 75% accuracy.^37^ The largest study addressed cardiovascular risk and included pain only as a feature contributing to the prediction.^3^ The second largest study, however, was closely related to pain research analyzing healthcare data from 392,492 patients with long-term back pain problems. Regression models, random forests, and support vector machines were combined with a transformer-based deep learning model to detect opioid use disorder in these patients.^22^

The often high classification performance of deep learning algorithms comes at the expense of large data sets, while random forests, support vector machines, classification and regression trees, k-nearest neighbors, Bayesian classifiers, and others perform quite well even with small data sets. Deep learning seems to be particularly useful in image analysis, where it can outperform the aforementioned algorithms, as several examples in the pain context above have shown. Of the 68 articles found for the present analyses in which deep learning was mentioned in the abstract, the sample sizes ranged from 2,170 to 2,766,434. However, the cohort sizes and the size of the analyzed data sets are not necessarily identical. For articles whose abstracts mentioned small sample sizes associated with deep learning, a full-text review generally indicated that the data sets used for training were imaging data of average sample sizes of 231,000 images. Generative adversarial networks however are examples for unsupervised deep learning involving the use of a discriminator next to a generator. This circumstance is a valuable advantage considering that the state-of-the-art GAN also involves data augmentation based on restructuring of the training data (eg, deep convolutional and conditional GAN) to handle smaller sample sizes.^95,97^

### 1.17. Geographical distribution of publication activities on machine learning in pain research

**Figure 6.**
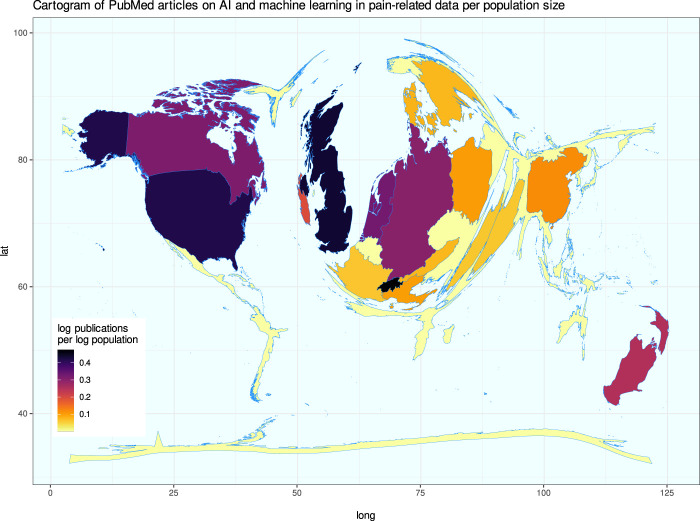
Bibliometric exploration of PubMed listed publications on the topic of AI and machine learning in pain research. Results of a computed PubMed database analysis of year and country of origin of publications not listed as reviews. Cartogram of the publication activity per county standardized at the average population of the respective country during the analyzed period, plotted as spatial plots with Gaussian blur as described in 24. Boundaries of regions are transformed to be proportional to publication counts. The figure has been created using the software package R (version 4.2.0 for Linux; https://CRAN.R-project.org/)^73^ and the libraries “ggplot2” (https://cran.r-project.org/package=ggplot2)^93^ and “Rcartogram” (https://github.com/omegahat/Rcartogram).^45^

The results of the above PubMed database search were further evaluated for bibliometric analyses on scientific activities in the field. The retrieved contributions were from 18 countries, according to the affiliation of the first authors, which was the determining factor for country assignment in the PubMed database. This may underestimate the joint contributions of other countries with the United States.^21^ Indeed, the United States occupies a prominent place in a cartogram^24^ of worldwide publications related to ML in pain research (Fig. 6), ie, a thematic map in which distortion is used to convey information, for example, by distorting the outline polygons of all countries in such a way that the areas are proportional to the number of publications. The United Kingdom, the Netherlands, Germany, Switzerland, Ireland, and New Zealand were also prominent source countries for contributions to ML and AI in the pain context.

## 2. Discussion

The use of machine learning methods in pain research is an accelerating trend worldwide, with the main area of application being human-derived and, in particular, patient data. This may indicate that these methods provide the necessary tools to directly address clinical pain and extract the desired knowledge from complex clinical information available in large data sets collected in the context of modern digital medicine. The present review of published articles in which pain is the immediate target of interest or is associated by the authors with the analysis of, for example, medical images, supports the view that machine learning is particularly efficient with large data sets; however, cohort sizes in pain-related studies peaking at n = 100 also suggest that the methods can be applied to smaller data sets. In particular, when the goal is not to train a clinically applicable automatic diagnostic tool but to discover knowledge, such as subgroup detection or selection of relevant information for subgroup assignment, a subset of machine learning methods has been shown to be efficient. Nevertheless, data needs are a limitation of machine learning, and the impact of using algorithms trained with patient data in clinical practice on patient care is receiving increasing attention among which the concept of “explainable AI”^1^ as one approach to the latter has been already applied in pain research.^16,54^

## 3. Limitations of machine learning methods

### 3.1. Data requirements

The limitations of ML are the large sample sizes that are often required, although the above analyses showed that cohort sizes around n = 100 were most common (refer to Fig. 5). One of the reasons why image-based analysis resulted in comparatively high classification performance, as in 43 where image diagnosis was performed with 97% accuracy while only 69% accuracy was achieved for image diagnosis, is the large amount of information contained in the image data. The present generic example of handwritten digits 0, …, 9 (Fig. 4) illustrates this. The digits were scanned by 1,797 people, and 64 pixels were determined for each digit, ie, 64 variables were available from each person, and the entire data matrix had the size of 115,008 data points. If the information was a pain score rather than images, the data set would contain only 1,797 data points. Thus, medical images are comprehensively captured by numerical information. By contrast, pain-related information is captured less comprehensively, often through pain scores and questionnaires selected to capture specific characteristics of pain according to the study objectives. Questionnaire sum scores provide even less information, and therefore, it may be better to analyze questionnaire data by item, as we have shown previously on psychological assessments in persistent pain.^55^

In addition to the requirements of often large data sets, supervised methods require labeled data, which is further increasing the costs for machine learning projects because labeling can be very expensive if performed by a physician. Medical diagnoses require time and often expensive additional testing such as imaging, laboratory markers, or various “omics” tests. A technical weakness is overfitting, which is not a particular feature of ML but occurs in basically all modeling approaches when a model is fitted to a data set such that it captures the current data almost perfectly but fails at generalization, ie, when applied to other data sets of the same type. When overfitting has occurred, the algorithm has “learned by rote” to assign a case to the correct class and therefore fails when a similar but not identical data set is addressed. Several precautions against this effect are standard procedures in training of algorithms, such as splitting the data into disjoint training and test subsets and permuting the training or test subsets.

### 3.2. Pathology dependency

Algorithms are limited to what they were trained with, ie, if an important facet of the problem was missing from the training data, or if a feature in the data set gained major importance during training but was otherwise of little interest, the algorithm may fail on future data although it seems to be technically successful. Unfortunately, it is not always quite clear what exactly the AI has learned. As mentioned in a related context,^57^ a classic and often-cited example is the fooling of neural networks trained to automatically detect camouflaged tanks in photographs of tanks in trees and photographs of trees without tanks.^17^ The network failed on a new set of photographs, and it turned out that it had been trained with photographs of camouflaged tanks taken on cloudy days while the new photographs had been taken on sunny days, and the neural network had learned to recognize the weather instead of distinguishing tanks from trees. The fact that machine learning algorithms only work with the information they have been trained with also implies that the exact same variables are needed when applied to a different data set unless they have been eliminated during feature selection, and new variables can only be included if the algorithms are re-trained. This also applies to the fact that when the algorithm is trained with imaging information, it is only applicable to imaging data. The shortcomings of this approach are not new because machine learning has been used, but it has also been clinically advisable in the past to base a medical decision not only on medical images but also by observing a broader spectrum of symptoms in the context of a patient.

Also related to the fact that algorithms are limited to the information used to train them is the difference between structural tissue changes and pain. This is true not only for peripheral morphological changes in osteoarthritis and low back pain, as shown in the examples above, but also for morphological differences in the brain. For example, social norms influence pain reporting behavior,^75^ and if this is not accounted for in data analysis, image-based training of algorithms may result in different accuracies of class assignment for pain reports although the same morphological information was addressed. However, this is not a particular technical weakness of ML but lies in the study design and the decision of which data are collected and included in the analysis. Similarly, it has been pointed out that physical pain can be easily inferred from a particular pattern of activated brain regions.^33^ This underscores that machine learning in the biomedical context, including pain research, should ideally be a concerted multidisciplinary effort to which computer scientists, biologists, physicians, and relevant other experts contribute with intensive discussions during project work.

### 3.3. Classification vs prediction

In many applications, machine learning provided classifications rather than predictions. In the sense of forecasting future events, the results are often reported as predictions. That is, the machine is trained to classify a case, eg, as “patient” or “healthy subject,” or as at risk versus not at risk. The algorithm does not predict risk directly but assigns a patient to the subgroup of patients at a certain risk. It should also be noted that algorithms when used as a classifier may need reevaluation over time. The importance of variables for class assignment may change over time as additional factors emerge that provide more relevant or modified information used for class assignment, or as factors interacting with the algorithm's target, such as pain, have undergone modulations that cause previously important variables, such as a genetic background, to become less important or to be modulated in their impact on the target. For example, genetic factors explained 63% of the variance in chronic pain at a first assessment in twins and 11% of the variance in chronic pain 12 years later.^7^ AE models based on paths were used for the analysis (Cholesky decomposition^4^) with “A” denoting additive genetic factors and “E” denoting environmental factors. In the second analysis performed on data on chronic pain acquired 12 years later, no additional factors to those found in the first analysis were found. By contrast, for nongenetic influences, environmental factors explained 37% of the variance in first assessment but 89% of the variance in chronic pain assessed 12 years later.

### 3.4. Differences between machine learning and statistics

**Figure 7.**
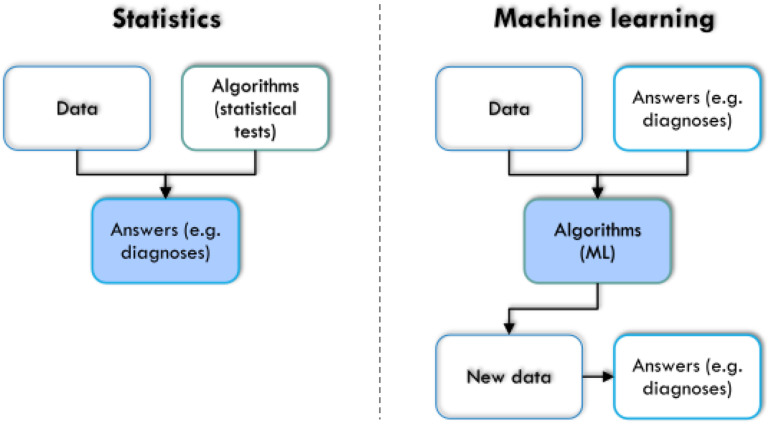
Simplified comparison between statistics and machine learning, based on the arrangement of the 3 elements “data,” “algorithms,” and “answers” to each other and on the final aim of the analysis (marked in blue). In statistics, algorithms are selected to be applied to the data to obtain an answer to a scientific question and that answer is the target of the analysis, whereas in machine learning, those answers may already be known and in its supervised form the trained algorithm is the target of the analysis. The figure was created using Microsoft PowerPoint (Redmond, WA) on Microsoft Windows 11 running in a virtual machine powered by VirtualBox 6.1.36 (Oracle Corporation, Austin, TX) as a guest on Linux and then further modified with the free vector graphics editor “Inkscape” (version 1.2 for Linux, https://inkscape.org/).

Although the usage of mathematical expressions is a combined feature shared by statistics and ML, both fields can be distinguished more or less. Statistical reasoning is built on an initial working hypothesis and focused on answering questions about the data based on descriptive exploration or induction using appropriate tests of significance. It analyzes whether the data sets come from the same distribution and were generated by the same data-generating process, or whether different processes underlie the observed data. Statistics decide whether experiments have a significant effect on the measured data based on a predefined hypothesis. In some contrast to this, ML is not necessarily dependent on a working hypothesis and is rather focused on answering questions referring to performance, about the data and model architectures, as well as about respective rules themselves. ML can be used to derive hypotheses from the data. It infers diagnostic capabilities or even discovers unknown structures from the data without bias, ideally leading to a better understanding of the underlying rules shaping the data. Moreover, for machine learning, the ability to assign cases to the correct classes is sufficient, and information from variables is crucial for this task that may even lack statistically significant differences between the classes; it has been shown that higher significance does not automatically mean greater relevance and variables important for correct class assignment may sometimes not significantly differ among classes.^49^ In a very simplified version (Fig. 7), statistics can be described as using data and algorithms, ie, statistical tests, to obtain answers about the research object of interest. Machine learning uses data and already available answers about the research object, eg, a previous classification, to obtain trained algorithms. Those can then be applied to new, unclassified cases to achieve a correct class assignment based on the characteristics of the cases. Thus, the outcome of statistics is an answer about the research object while the result of (supervised) ML is a trained algorithm. On that basis, deeper information about the data can be exploited. Subsequent learning about the research subject has been shown in several examples in this report (refer to, for example, the chapter on feature selection or the remarks on knowledge discovery or XAI).

### 3.5. Role of machine leaning in medical diagnostics and pain treatment

Machine learning and “AI” are currently often presented together with the expectation that these methods will take over certain tasks from physicians. Ethical and legal challenges of artificial intelligence–driven health care are the subject of much discussion in lay and scientific forums; an example of a publication by that very name is 25. This report is not intended to contribute to this discussion because its focus is on the review and context of machine learning in pain research. Nevertheless, a few points may be mentioned. First, trustworthiness must be considered both in the sense of demonstrably correct functioning of AI for medical (diagnostic) tasks and in the sense of transparency of the decision, as addressed in the concept of explainable AI (XAI).^1,52^ Second, final medical decisions should remain with the physician, and even their critical discussion and correction requires a human interacting with the patient, not a robot. However, AI can be of great value to both physicians and patients if, for example, it takes over repetitive medial tasks, improves diagnosis, or makes treatment suggestions based on evidence and likelihood of success. One successful approach may be to combine artificial and human intelligence, as recently demonstrated in diagnosing breast cancer from radiographies, where the combination was better than diagnoses made by either physicians or AI alone.^47^

## 4. Conclusions

Machine learning applications to pain have increased dramatically in the last decade, with more than half of the publications in recent few years. The current enthusiasm for ML and AI must be accompanied by a careful consideration of the shortcomings of these methods,^36^ of which some have been highlighted in this report. Nevertheless, the correct application of the right methods to the right data is a pervasive principle in science that is not specific to ML. However, at the current, early stage, ML methods are less well known than classical statistics, which places additional demands on the review process of scientific reports. AI and ML are being used in a broader context that is also relevant to pain. This includes their application in drug discovery and development and other areas of pharmacological research. In addition, AI techniques are being used in virtual reality approaches that have been introduced in recent years in the scientific development of novel approaches to pain management (for a review, refer to 63). ML is not limited to training and applying classifiers as biomarkers or other diagnostic tools. An important application is the discovery of structures in the data that directly addresses complex pain phenotypes, the properties characterizing these phenotypes and their underlying complex mechanisms including omics information and others. The present scientometric analysis was generated using programmed information mining and human experts with biomedical, biological, and data science backgrounds. The combination of artificial and human intelligence is probably among the most promising approaches for the advancement of pain research.

## Disclosures

The authors have no conflicts of interest to declare.
